# Regulation of Pre-mRNA Splicing: Indispensable Role of Post-Translational Modifications of Splicing Factors

**DOI:** 10.3390/life13030604

**Published:** 2023-02-21

**Authors:** Miroslava Kretova, Tomas Selicky, Ingrid Cipakova, Lubos Cipak

**Affiliations:** Department of Genetics, Cancer Research Institute, Biomedical Research Center, Slovak Academy of Sciences, Dubravska cesta 9, 84505 Bratislava, Slovakia

**Keywords:** pre-mRNA splicing, splicing factors, alternative splicing, post-translational modifications, gene expression

## Abstract

Pre-mRNA splicing is a process used by eukaryotic cells to generate messenger RNAs that can be translated into proteins. During splicing, the non-coding regions of the RNAs (introns) are removed from pre-mRNAs and the coding regions (exons) are joined together, resulting in mature mRNAs. The particular steps of splicing are executed by the multimegadalton complex called a spliceosome. This complex is composed of small nuclear ribonucleoproteins, various splicing factors, and other regulatory and auxiliary proteins. In recent years, various post-translational modifications of splicing factors have been shown to contribute significantly to regulation of processes involved in pre-mRNA splicing. In this review, we provide an overview of the most important post-translational modifications of splicing factors that are indispensable for their normal function during pre-mRNA splicing (i.e., phosphorylation, acetylation, methylation, ubiquitination and sumoylation). Moreover, we also discuss how the defects in regulation of splicing factors are related to the development of cancer.

## 1. Introduction

Pre-mRNA splicing is a process characterized by the cutting out of non-coding sequences (introns) and joining the coding sequences (exons) together to produce mature mRNAs. Introns in the pre-mRNAs consist of several conserved sequences important for splicing, including 5′ and 3′ sites of splicing and the 18 to 40 base pairs long branch point sequence (BPS), located upstream of the 3′ splice site. Introns also contain the polypyrimidine tract (PPT), which is critical for recruiting splicing factors to the 3′ splice site [1].

During splicing of the pre-mRNA, two transesterification reactions remove introns from pre-mRNA. First, the 5′ site of splicing is cleaved and the 5′ end of the intron is ligated to the adenosine within the BPS. Next, the 5′ and the 3′ ends of exons are joined together after the 3′ site of splicing is cleaved by the 3′ OH group of the 5′ end of exon [2]. These reactions are catalyzed by the multimegadalton complex, spliceosome. The spliceosome is composed of the small nuclear ribonucleoproteins (snRNPs) U1, U2, U4, U5 and U6, which are part of a major spliceosome, or five snRNPs, U11, U12, U4atac, U5 and U6atac, which form a minor spliceosome. Additionally, numerous splicing proteins and factors, like those forming the NTC and NTR complexes, the RNA-dependent helicases and other regulatory proteins, are part of the spliceosome [3,4,5,6].

**Figure 1:**
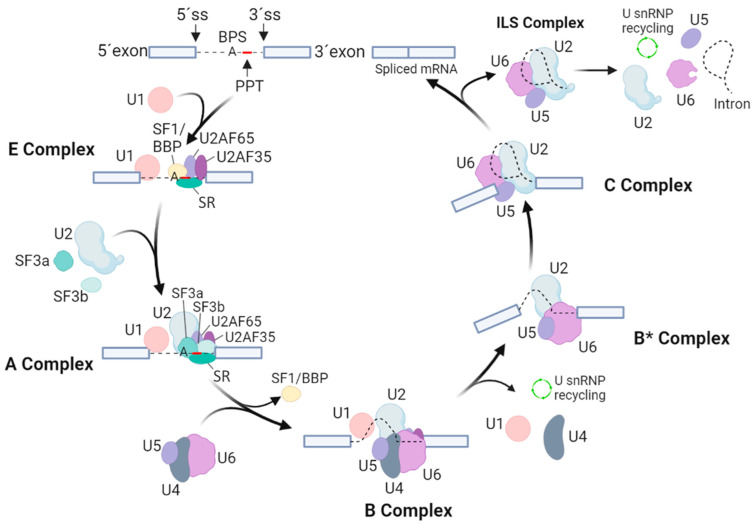
Overview of spliceosomal assembly, rearrangements and catalytic cycle. The scheme indicates the particular splicing factors and subcomplexes they form. Exons are marked by cool grey boxes, while thin black lines imply for intron and intron lariat. U1, U2, U4, U5 and U6 stand for small nuclear RNPs (snRNPs), SR represents the proteins of the serine–arginine-rich family, 5′ SS and 3′ SS sites represent 5′ and 3′ sites of splicing, PPT is the polypyrimidine tract, and BPS points to a branch point sequence.

Assembly of the spliceosome starts with the ATP-independent binding of the U1 snRNP through base-pairing interactions of the 5′ end of the U1 snRNA to the 5′ splice site of the intron. This interaction is supported by proteins of the serine–arginine-rich (SR) family and by the U1 snRNPs. The U1-5′ splice site interaction also involves the binding of the SF1/BBP and the U2AF to the BPS and the PPT [3]. Next, the 3′ splice site is bound by two specific U2 auxiliary factors, U2AF65 and U2AF35. The U2AFs interact directly with the polyA cleavage machinery [7,8], while the U1 snRNP interacts with nascent RNA to ensure polyA cleavage, polyadenylation and transcription elongation [9,10,11,12]. The SF1/BBP protein then interacts with U2AF65 through its C-terminal RNA recognition motif. Next, the U2AF35 of the U2AF heterodimer locates to the AG dinucleotide of the 3′ splicing site. Together, these steps lead to an assembly of the spliceosomal E complex, which ensures the initial recognition of splice sites of the intron (Figure 1).

After the assembly of the E complex, the U2 snRNA is enrolled in an ATP-dependent manner in a base-pairing interaction with the BPS. This allows the formation of the A complex. The U2 snRNPs, like SF3a and SF3b, and the U2AF65 factor then stabilize this complex [13,14]. The presence of the U2 snRNA then results in dissociation of SF1/BBP from the BPS. Next, SF3b14a/p14 binds to adenosine within the BPS, and SF3b155 is replaced with the U2AF65 [15,16]. Then, the preassembled U4/U6-U5 tri-snRNP is bound, leading to formation of the B complex. Formation of the U4/U6-U5 tri-snRNP is further promoted by the TSSC4 protein, which is a part of this complex [17]. Although all necessary snRNPs are within the B complex, this complex is still inactive and requires additional rearrangements to be competent for the first splicing step. To activate the B complex, U1 and U4 snRNPs need to be released. The activated B* complex then executes the first catalytic step of splicing, generating the C complex. Next, the C complex undergoes rearrangements to allow the second catalytic step, assembling into the post-catalytic spliceosome (P complex) that contains the lariat intron and spliced exons [18]. After the second catalytic step, the mRNA is released from the P complex, resulting in the formation of the intron lariat spliceosome (ILS complex). Finally, the U2, U5 and U6 snRNPs are released to be reused for next round of splicing [3].

## 2. Alternative Splicing and Transcriptional Level Expansion

The concept of alternative splicing was first introduced in 1977, when introns and exons were discovered in the adenovirus *hexon* gene. It was suggested that various arrangements of exons could give diverse mRNA isoforms [19,20], thus increasing the functional diversity of the proteome [21,22]. As such, alternative splicing has become accepted as a post-transcriptional regulatory mechanism to explain how proteome diversity can be achieved with a limited set of genes [23,24]. However, some recent studies questioned the proteome diversity prominent expansion by alternative splicing at the protein level [25,26,27]. Additionally, it was proposed that alternative splicing might simply represent the noise in the splicing machinery. This was supported by studies demonstrating that the majority of genes express single dominant isoforms represented at the protein level [28,29]. Despite this, alternative splicing has become accepted as a mechanism that significantly contributes to transcriptional level expansion to allow the cells to increase the proteome complexity [30]. However, our understanding of how and to what extend the alternative splicing affects proteome diversity is still far from clear. Therefore, further studies are needed to support the role of alternative splicing for transcriptomic and potentially proteomic variations.

Recently, it has been established that up to 95% of human multiexon genes are alternatively spliced to encode proteins with different functions [31]. In contrast, in yeasts, the ratio of alternative splicing events ranges from 0.2% in the budding yeast *S. cerevisiae*, which contains introns in ~3% of its genes, to ~3% in the fission yeast *S. pombe*, in which about 43% of genes contain introns [32,33,34].

Many studies have shown that splicing factors cooperate to produce the corresponding transcripts from particular genes. Normally, splicing factors bound at the 5′ and the 3′ ends of an intron interact with each other as a part of spliceosome assembly. However, it was found that a downstream 5′ site of splicing might activate a weak 3′ site of splicing located upstream of an exon. This also suggested that the molecular interactions across an exon might define the site of splicing [35]. Importantly, it was shown that the recognition of splice sites was affected by the RNA binding proteins (RBPs). Particularly, the serine–arginine-rich (SR) family of proteins was found to play an important role in the recognition of splice sites. These proteins were found to bind to exonic splicing enhancers (*ESEs*) in pre-mRNA to recruit the splicing machinery to the splice site [36,37,38]. Additionally, the SR proteins were shown to regulate other aspects of gene expression. They partially overlapped but had a distinct role in transcription-coupled splicing and processing of pre-mRNAs. Furthermore, some shuttling SR proteins were assigned as the adaptors to export mRNAs into the cytoplasm [39].

Additionally, many transcription activators, like class I, class IIA and class IIB, play an important role in alternative splicing [40,41]. It is worth mentioning that promoters and enhancers regulate expression of genes by employing the protein–DNA and protein–protein interactions. It was revealed that promoters assign the place for RNA polymerase, while enhancers and promoters control initiation of transcription and elongation. This supports the concept of mutual interconnections between transcription and splicing [42,43,44,45].

Currently, there are seven identified modes of alternative splicing: cassette alternative exon, alternative 5′ site of splicing, alternative 3′ site of splicing, intron retention, mutually exclusive alternative exons, alternative promotor and first exon, and alternative polyA site and terminal exon [30,46]. These types of splicing commonly occur together, forming more than 25% of alternative splicing events. Interestingly, alternative splicing may happen in the translated as well as in the untranslated regions of the transcripts [47].

## 3. Importance of Cross-Communication between Splicing and Transcription

Transcription and splicing are processes that depend on protein–DNA, protein–RNA, and protein–protein interactions. It was found that splicing occurs in the close vicinity of genes and is usually co-transcriptional. Both processes, splicing and transcription, are coordinated and, in many cases, functionally coupled [45]. A comprehensive proteomic analysis of the human spliceosome has found that more than 30 out of the 145 spliceosomal factors are known, or are candidates for coupling the splicing to other gene expression steps [48,49]. It has been also shown that transcription elongation and splicing can be influenced reciprocally. Elongation rate can control splicing, and splicing factors then regulate RNA polymerase II elongation [41,45,50].

It has been found that the nascent RNAs undergo splicing during transcription elongation, immediately after the RNA polymerase II completes transcription [51,52,53]. The C-terminal domain (CTD) of RNA polymerase II is involved in gene-expression-related functions, including the 5′ capping, splicing, polyadenylation and chromatin remodeling [54]. The CTD of RNA polymerase II has a vital role in RNA processing. It has been found that it acts as a “landing pad” for sequestering other splicing factors. In mammals, the CTD consists of 52 tandem repeats of the heptapeptide YSPTSPS, which help to recruit splicing factors to the nascent RNA transcripts [55]. In particular, phosphorylated CTD of RNA polymerase II could attract the splicing factor U2AF2, which then helps to bind PRPF19 and U2AF2 to the nascent pre-mRNA [56]. It has been found that snRNA components also associate with RNA polymerase II when its CTD is phosphorylated on S5 of its YSPTSPS repeats [52]. This phosphorylation is linked with transcription initiation through activity of CDK7. On the other hand, phosphorylation on S2 of the YSPTSPS repeats is preferentially linked with the CTD of RNA polymerase II activity at the 3′-end of genes through cyclin-dependent kinase 9 of the transcription elongation factor b [57]. In addition, phosphorylation on S7 of the YSPTSPS repeats has been found to be important for elongation and splicing [58]. Thus, phosphorylation of the CTD of RNA polymerase II represents a mechanism that brings together transcription and splicing processes [59]. Importantly, whole genome and transcriptome sequencing studies have proved the high complexity of splicing regulation and have shown that splicing occurs co-transcriptionally and is influenced by chromatin status and mRNA modifications [60].

## 4. Role of Post-Translational Modifications in Regulation of Splicing Factors

**Figure 2:**
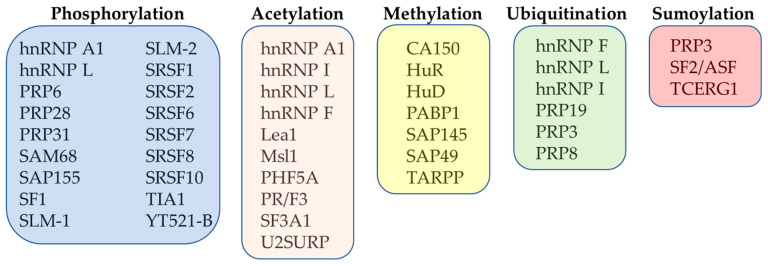
Overview of the most important PTMs of selected splicing factors.

Different post-translational modifications (PTMs) have been found to be involved in the structural rearrangements of ribonucleoprotein particles and spliceosome-associated factors [61]. The PTMs have been found to contribute significantly to regulation of splicing and to its integration into regulatory networks controlling expression of genes in eukaryotes [62]. As such, more attention has been given to studies that have focused on better understanding of the role of PTMs in the context of pre-mRNA splicing regulation. The most important PTMs that regulate the function of splicing factors are represented by phosphorylation, acetylation, methylation, ubiquitination and sumoylation (Figure 2).

### 4.1. Phosphorylation

Dynamic phosphorylation of the spliceosomal proteins and accessory splicing factors was shown to be crucial for the correct coordination of both constitutive and alternative splicing. It was shown that phosphorylation is of great importance for the proper assembly and activation of many factors forming the spliceosome, including PRP6, PRP28 and PRP31 [63,64]. Particularly, the activity of hPrp28 was found to be regulated through phosphorylation by SRPK2 [63]. Similarly, the activities of U5 and U2 snRNP components, U5–156 kDa and SAP155, were shown to be regulated by their dephosphorylation [65,66,67]. Furthermore, PP1 and PP2A phosphatases were shown to be required for the catalytic steps of the splicing reaction through dephosphorylation of many spliceosomal proteins [65,68,69].

Phosphorylation and dephosphorylation thus provide a fine regulation of functions of the spliceosomal components and RBPs during selection of the sites of splicing. For instance, the SF1 and SRSF1 phosphorylation regulates their binding with the U1 snRNP and the U2AF65, thus affecting the spliceosome assembly [70,71,72,73,74,75,76]. Phosphorylation of SR proteins was also found to be important for their regulation [77]. It was observed that phosphorylation of the RS domain of these proteins is involved in regulation of their protein–protein and RNA–protein interactions [78,79,80,81,82]. Another factor that was found to be regulated through phosphorylation is SRSF10. The SRSF10, when phosphorylated on its RS domains, works as a specific activator of splicing [83,84]. It was also found that phosphorylation of the SRSF10 is required for the proper assembly of U1 and U2 snRNPs on pre-mRNAs [83]. However, under stress conditions, the SRSF10 was found to be rapidly dephosphorylated by PP1 phosphatase [85].

Phosphorylation is very important also for the regulation of activity of other splicing factors. It was found that during stress conditions the hnRNP A1 is phosphorylated by the MAPK p38. This factor is then translocated into cytoplasm to cooperate on alternative splicing [86,87]. Phosphorylation also affects the splicing activity of hnRNP L, as it reduces the interaction of the hnRNP L with the U2AF65 subunit of the U2AF [88,89].

Some splicing factors, like SAM68, can be influenced by phosphorylation [90]. It was found that Tyr phosphorylation by SRC-family kinases (SFKs) causes the accumulation of SAM68 nuclear bodies [91,92,93]. Moreover, it was observed that phosphorylation of the SAM68 disturbed its binding with the hnRNP A1 to the *BCL-X* pre-mRNA, thus diminishing its splicing [93]. Furthermore, the phosphorylation of the SAM68 by ERK1/2 was found to potentiate splicing of the variable exons in the *CD44* [94,95,96].

Phosphorylation of splicing factors, except by the above-mentioned kinases, is mediated also by specific splicing kinases. These kinases are important regulators of the splicing process. They are represented by the SRSF protein kinases (SRPKs), the pre-mRNA splicing 4 kinase (PRP4K/PRPF4B) and the CDC-like kinases (CLKs). These kinases phosphorylate numerous spliceosomal factors and SR proteins, thus regulating their localization within nuclear speckle domains, as well as their transport from nucleus into the cytoplasm [97].

It was found that SRPKs phosphorylate SR proteins both in the cytoplasm and in the nucleus [62]. Phosphorylation of SR proteins in the cytoplasm was shown to be important for their nuclear import, as the phosphorylation enhances interactions of SR proteins with their specific import receptor, transportin SR2 [98,99]. Several reports suggested that SRPK1 mediates nuclear localization of SRSF1 by phosphorylating its RS domain [74,75,76,100,101,102]. Phosphorylated SRSF1 then defines the processing of splice sites by interacting with corresponding RNPs and *ESE* sequences [103,104,105].

PRP4K/PRPF4B is a Ser/Thr kinase identified during the screening for splicing defects of temperature-sensitive mutants of *S. pombe* [106]. The mammalian PRP4K kinase was found to form a complex with splicing factors PRP6, U5 snRNP and SRSF8 [64,107]. Furthermore, this kinase was demonstrated to phosphorylate PRP6 and PRP31, thus helping with assembly of U4/U6-U5 tri-snRNP [64].

CLKs are a family of SR protein kinases characterized by the C-terminal kinase domains with dual specificity, and the N-terminal RS domains important for binding to SR proteins. These kinases were found to phosphorylate SR proteins, thus regulating their activities and localization within nuclear speckles [108]. The concerted action of CLKs and SRPKs was shown to be important for regulation of the splicing process. For example, it was observed that IGF-1- and TNF-*α*-induced production of pro-angiogenic VEGFxxx isoforms depends on the activation of SRPKs, whereas TGF-*β*1-enhanced antiangiogenic isoforms VEGFxxxb production depends on the activity of CLKs [109,110].

Other kinases, known as signaling-activated kinases, were found to affect the splicing by phosphorylating the splicing factors and their regulators [62]. It was reported that AKT kinase regulates the function of many RBPs. This kinase was shown to phosphorylate both SR proteins and hnRNPs, affecting their splicing-dependent and splicing-independent functions [111]. It was shown that AKT-dependent phosphorylation of hnRNP L supports its binding to exon 3 of *CASPASE-9b*, thus inducing expression of this anti-apoptotic splicing variant. Additionally, AKT-phosphorylated hnRNP L was found to compete with hnRNP U. This led to an impairment of hnRNP U and a decreased level of the pro-apoptotic splicing variant CASPASE-9a [112]. Moreover, AKT kinase was found to affect the translational activity of hnRNP A1. When phosphorylated by AKT, this factor loses its ability to support IRES-dependent translation of the *cMYC* and the *CCND1* [113]. Similarly, AKT-dependent phosphorylation of SRSF1 and SRSF7 potentiated the inclusion of the *EDA* exon in the fibronectin mRNA, thus supporting the translation of this variant [114]. AKT was also found to have a role in EGF-induced splicing through its binding to SRPKs, and inducing their autophosphorylation and dissociation from the HSP70 chaperone. This results in an enhanced SRPKs nuclear translocation guided by HSP90 and phosphorylation of SR proteins [102].

MAPKs are a family of Ser/Thr kinases that also participate in the tuning of splicing. The MAPKs were shown to regulate the alternative splicing of the *CD44* through the RAS-RAF-MEK-ERK signaling pathway [115,116]. SAM68 was identified as a substrate of this pathway. This factor was found to interact with the splicing factor U2AF65 and enhance the recognition of the 3′ site of splicing. Phosphorylation of SAM68 reduced the ability of the SAM68/U2AF65 complex to bind *CD44* pre-mRNA [94]. Similarly, ERK-dependent phosphorylation of SAM68 enhanced its affinity to the 3′ UTR of the SRSF1 transcript. This allowed intron retention and generation of a full-length SRSF1 transcript, thus blocking alternative splicing events. These are known to downregulate levels of SRSF1 transcripts by directing them to degradation by a nonsense-mediated mRNA decay pathway [117,118]. Furthermore, other MAPKs, such as p38 and JNKs, were also found to phosphorylate and modulate the activity of splicing factors. Phosphorylation of SPF45 was shown to abrogate SPF45-dependent inclusion of exon 6 in the *FAS*. This resulted in expression of its dominant negative isoform that inhibits FAS-mediated cell death [119,120].

Fas-activated serine/threonine kinase (FAST) is a constitutively phosphorylated kinase. This kinase is known to undergo a rapid dephosphorylation after the binding of Fas ligand to its receptor. It was shown that the dephosphorylated form of FAST kinase might phosphorylate the TIA1, thus enhancing U1 snRNP recruitment to *FAS* pre-mRNA. This supports the recognition and inclusion of the variable exons of *FAS* into its mRNA, including exon 6 [121,122]. Furthermore, the FAST kinase was also found to regulate the splicing of *FGFR2*, favoring the inclusion of exon III b in its mRNA [123].

Another Ser/Thr kinase that has been identified as being involved in the regulation of splicing is Aurora Kinase A (AURKA). This kinase was shown to participate in splicing of anti-apoptotic BCL-XL by stabilizing the SRSF1. Cells with diminished activity of AURKA had lower levels of SRSF1 and alleviated levels of pro-apoptotic BCL-XS [124,125].

Protein tyrosine kinases (PTKs) are other kinases involved in regulation of splicing. It has been shown that these kinases affect gene expression at the level of alternative splicing. The regulated splicing factors account of RBPs such as the members of the STAR protein family, in particular SAM68 [62]. For example, breast tumor kinase (BRK) is a nonreceptor tyrosine kinase (nRTK) that phosphorylates SAM68, SLM-1 and SLM-2 and reduces their RNA binding [126,127]. It was found that SAM68 is a target of FYN, another nRTK. FYN-dependent phosphorylation of SAM68 prevented SAM68-dependent splicing of the *BCL-X* and *CCND1*. This phosphorylation lowered the affinity of SAM68 to RNAs and abrogated its binding with the hnRNP A1 [93,128]. Phosphorylation also influenced the activity of splicing factor YT521-B, which is known to be regulated by several nRTKs. Phosphorylated YT521-B was shown to regulate the selection of splice sites of different genes [129,130].

The cAMP-dependent protein kinase (PKA) was found as a kinase that colocalizes with SRSF2 into the nucleus, and phosphorylates SR proteins to regulate alternative splicing [131,132]. It was revealed that an increased level of intracellular cAMP can modulate the alternative splicing events through phosphorylation of hnRNPs and SR proteins by PKA [62]. The bioactive small molecule compound forskolin, which is an adenylyl cyclase activator, was found to affect the efficiency of splicing of exon 10 of the *TAU* by phosphorylating the splicing factors SRSF1 and SRSF7 by PKA [133,134,135]. PKA also affected the splicing of neuronal differentiation genes by phosphorylating hnRNP K. Phosphorylated hnRNP K was found to have a stronger binding activity to mRNAs, leading to inaccurate recognition of the 3′ sites of splicing [136].

Another protein kinase that is involved in regulation of SR proteins is dual specificity tyrosine phosphorylation-regulated kinase 1A, DIRK1A. It was found that the DIRK1A colocalizes with the SRSF2 in nuclear speckles. Overexpression of this kinase was shown to promote the disassembly of nuclear speckles [137]. It was also found that the DIRK1A phosphorylates the SRSF1 and SRSF7 or SRSF2 and SRSF6 to induce their cytoplasmic translocation or dissociation from nuclear speckles, respectively [135,138,139,140]. This seems to be the main mechanism by which DIRK1A kinase regulates the activities of these SR proteins [62] (Table 1).

Finally, some regulatory proteins also possess kinase activity towards splicing factors. For example, DNA topoisomerase I was shown to phosphorylate SR proteins [141]. Deficiency in DNA topoisomerase I was found to cause the hypophosphorylation of SR proteins. This resulted in dysregulation of the alternative splicing [79]. Additionally, treatment of cells with a specific inhibitor of DNA topoisomerase I resulted in a reduced phosphorylation level of SR proteins, which in turn abolished the assembly of the spliceosome and affected the splicing efficiency [142]. Thus, the double activity of DNA topoisomerase I might represent the regulatory mechanism that ensures the correct coordination between splicing and DNA transcription [143].

### 4.2. Acetylation

Many studies have shown that the rearrangements that take place at the time of co-transcriptional spliceosome assembly are connected with specific histone modifications [144,145]. It is known that genomic DNA is packed in chromatin fibers within the nucleosomes. These are composed of two copies of each of the four histone proteins H2A, H2B, H3 and H4 [146]. Histones undergo extensive PTMs on their N-terminal tails. These modifications influence compaction of DNA and allow binding of specific regulatory factors. One of the most important histone PTM linked to transcriptional regulation is H3 and H4 lysine (K) acetylation [147].

Several studies suggested that nucleosomes and specific modifications of histones were enriched on exons and might represent certain type of histone “marks” that could be linked to splicing signals [148,149,150,151]. Similarly, proteins that were located close to methylated histones (H3K4me3 and H3K36me3) were found to help recruit the snRNPs to the nascent transcript, thus affecting the efficiency of splicing [152,153]. In yeast, H3K36me3 was found to be significantly enriched on exons of the transcribed genes [154]. Furthermore, the H3K36me3 was found to facilitate the binding of histone deacetylases (HDACs), the enzymes that deacetylate histones to control the transcription [155,156,157]. Moreover, PSIP1 was shown to bind to H3K36me3 to help recruit the SRSF1 [158]. Additionally, SUMO-1 marks were found to correlate with H3K4me3, and reduction of E2 factor UBC9 or SUMO-1 resulted in lower levels of mRNA of genes encoding ribosomal protein subunits and translation regulatory factors. This indicated that these genes were still transcribed but at lower levels [89]. These findings suggest that PTMs of histones are important for the regulation of pre-mRNA splicing.

Similarly, as for phosphorylation, many splicing factors were found to be regulated by acetylation [159]. It was found that histone acetyltransferase Gcn5 was essential for binding of the U2 snRNP to BPS. Dysregulation of histone acetylation by mutating the lysines of histone H3, or deactivating the Gcn5, was found to be destructive when combined with deletion of U2 snRNPs, such as Msl1 and Lea1 [160].

HDACs play important roles in modulating gene transcription, chromatin structure and splicing [161,162,163,164]. It has been found that many HDACs also deacetylate non-histone proteins. This suggests the role of HDACs in non-chromatin/transcription-related processes [165]. While the association between RNA splicing and histone modifications has been widely accepted, recent evidence reveals that HDACs can bind to spliceosomal and ribonucleoprotein complexes [145]. It was found that HDAC1, HDAC2 and SRSF1 regulate the splicing of the *MCL1* [166,167]. HDACs also regulate the alternative splicing by stabilizing the hnRNPs. It was found that more than 85% of hnRNPs contain at least one lysine prone to PTMs. For example, hnRNP F contains K87, K98 and K224 residues that were found to undergo acetylation or ubiquitination. Similarly, several lysines of hnRNP A1, I and L were found to be acetylated/ubiquitinated. This finding suggested that the stability of the hnRNPs involved in alternative splicing might be controlled by acetylation and ubiquitination [168].

Furthermore, identification of non-histone HDAC substrates after inhibition of SIRT1 deacetylase led to identification of splicing factors regulated by acetylation, such as PRPF3, SF3A1 and U2SURP [169,170,171,172,173]. Interestingly, pre-mRNAs encoding many non-histone HDAC substrates were also found to be subjected to regulation by alternative splicing [165]. For example, the function of P53 protein was found to be regulated by HDACs [174,175,176]. Furthermore, the deactivation of *SRSF1* and *SRSF3* led to upregulation of *P53β* [177,178]. Recently, K29 acetylation of PHF5A was found to be induced by multiple cellular stresses. Alteration in the acetylation of PHF5A affected the global pre-mRNA splicing, including the promotion of KDM3A protein expression through pre-mRNA alternative splicing [179].

### 4.3. Methylation

Methylation is a post-translational modification that functions primarily in cell signaling and cell fate decisions [180,181,182,183]. However, recent studies showed that methylation might also be involved in regulation of pre-mRNA splicing [184,185,186,187,188,189]. Methylation is executed by members of the PRMT family and the putative arginine methyltransferase NDUFAF7. PRMTs catalyze the binding of a methyl group from S-adenosylmethionine to the guanidino nitrogen atoms of arginine, making methylarginine and S-adenosylhomocysteine [190,191,192].

It was shown that arginine methylation is required for maturation of snRNPs [182,193,194,195]. For example, SAP145 and SAP49 were found to bind to PRMT9, thus linking this methyltransferase to maturation of the U2 snRNP [195]. It was also found that PRMT9 can monomethylate and symmetrically di-methylate the spliceosome-associated protein SAP145 [187,195]. This splicing factor binds to the BPS and has an important role in the early steps of splicing [196]. Additionally, PRMT9 was shown to be a modulator of the SAP145/SAP49 complex that plays a role in snRNP maturation in the cytoplasm. It was also observed that methylation of SAP145 on R508 is required for creation of a binding site for the SMN protein, and an alteration of the PRMT9 level led to global splicing defects [195]. The Tudor domains of SMN, SPF30 and TDRD1/2/3/6/9/11 were identified as the main methylarginine-interacting domains [197,198].

Moreover, methylation of SR proteins and hnRNPs was found to be important for regulation of their subcellular localization [199,200,201,202]. In the context that pre-mRNA splicing is frequently co-transcriptional, the elongation factor CA150 was detected to be methylated by PRMT4/CARM1 and PRMT5, thus promoting exon skipping [193]. In vitro methylation assays further confirmed that splicing factors CA150, SmB, U1C and SAP49 are specifically methylated by CARM1 [194]. Searching for novel CARM1 substrates led to an identification of the RNA-binding HuR and HuD proteins, PABP1 and TARPP, chromatin remodeling proteins, histone H3 and p300/CBP [203,204,205,206,207]. Thus, the capacity of CARM1 to methylate RBPs pointed to the role of methylation in alternative splicing regulation [208].

### 4.4. Ubiquitination

Ubiquitination is an important type of post-translational modification that affects the stability, localization and activity of proteins [209,210,211]. Besides its function in regulation of various cellular processes, recent studies proposed that ubiquitination is also important for the regulation of spliceosome assembly. For instance, the splicing factors Prp3, Prp19, Usp4 and Prp24/Sart3 were reported to participate in poly-ubiquitination cycle. It was reported that Prp19 has a U-box that allows its self-ubiquitination through nonproteolytic K63-linked chains [212]. Later, it was shown that ubiquitination and deubiquitination of Prp8 might regulate U4/U6 unwinding required for activation of the spliceosome [213]. It was also demonstrated that the Prp19 complex is in fact E3 ubiquitin ligase that ubiquitinates Prp3, thus allowing its interaction with Prp8 [214]. Interestingly, a recent study revealed that ubiquitination on the K1246 residue within the catalytic domain of RNA polymerase II links the slower elongation and transcriptional pausing to pre-mRNA splicing. It was found that RNA polymerase II deubiquitination by Bre5-Ubp3 ubiquitin protease complex allows the elongation to resume [215]. 

Another example of the involvement of ubiquitination in regulation of splicing processes is the interaction between an atypical ubiquitin-like protein UBL5/Hub1 and the spliceosome. It was found that UBL5/Hub1 binds to the spliceosome by interacting with splicing factors Snu66, Prp38 and Spp381, or with DEAD-box RNA helicase Prp5 [216,217,218,219]. Structural analyses of UBL5/Hub1 complexes revealed that this protein is a part of several spliceosomal complexes, particularly precatalytic and activated spliceosomal complexes [220,221,222].

It was also demonstrated that UBL5/Hub1 makes a direct contact with PRPF8/Prp8 [221,222]. Interestingly, UBL5/Hub1 was found to interact with Snu66 in the spliceosomal B complex and with Spp381 within the spliceosomal B* complex. Together with Spp381, this protein stabilizes the position of the 5′ site of exon and helps to anchor the U5 snRNA into precatalytic and activated spliceosomal complexes [221]. It has been shown that UBL5/Hub1 interacts also with an evolutionarily conserved RNA helicase of the DEAD-box protein family DDX46 [218]. This helicase was found to hold together U1 and U2 snRNPs during early steps of spliceosome assembly [223,224]. In yeast, Hub1 was found to bind to and modulate the splicing activity of Prp5. Interestingly, overexpression of Hub1 affected the selection of sites of splicing, most likely due to faster splicing kinetics of Prp5 [218]. Recently, it was found that Hub1-mediated alternative splicing also has an important role for stabilizing the nuclear envelope [225]. It was revealed that Hub1 participates in alternative splicing of *SRC1,* which encodes an inner nuclear envelope protein [216,226]. Importantly, cells lacking Hub1 were not able to express the Src1-S isoform due to a deficiency of splicing upstream of the corresponding splice site [216].

Moreover, UBL5/Hub1 was also found to play a role in the regulation of sister chromatid maintenance. It was shown that a deficiency in UBL5 causes a global reduction in efficiency of splicing [217,227]. Notably, it was found that cells deficient in UBL5 were unable to splice the first intron of *Sororin*, the gene encoding a cohesion protection factor. This resulted in a significant loss of the Sororin level leading to its decreased binding onto chromatin, and premature sister chromatid separation [227].

### 4.5. Sumoylation

Sumoylation is a type of post-translational modification that has been found to be involved in regulation of pre-mRNA splicing processes, such as the pre-mRNA 3′ end processing, RNA editing, RNA binding by hnRNP proteins and mRNA packaging into mRNPs [228,229,230,231,232].

The first evidence for an involvement of sumoylation in regulation of splicing came from the finding that SUMO E3 ligase PIAS1 is a part of the spliceosome [233]. Moreover, the nuclear bodies, such as speckles and Cajal bodies, which are enriched for splicing factors, were found to contain SUMO pathway components [234,235,236]. It was also reported that many splicing factors are in a SUMO-conjugated form during splicing. The same is true for the splicing factor Prp3, which is important for the formation of U4/U6-U5 tri-snRNP and activation of the spliceosome [237,238].

In addition, it was shown that splicing factor SRSF1 might regulate sumoylation by affecting activity of PIAS1. SRSF1 overexpression or depletion disturbed the level of SUMO conjugation to different spliceosomal proteins, including Prp3, Prp28, Snu114 and U2AF65. Interestingly, it was found that sumoylation-deficient Prp3 was able to interact with U4/U6 snRNA. However, its ability to bind to U2 and U5 snRNPs was impaired. This affected spliceosome activation and compromised the efficiency of splicing [237]. This finding suggested that the splicing factor SRSF1 might be indirectly involved in the regulation of sumoylation of other splicing factors [237,239,240].

Sumoylation can also repress transcription. It was found that sumoylation of TCERG1 impaired its elongation stimulatory activity, while mutation of the SUMO acceptor lysines of TCERG1 potentiated the elongation rates [241]. Similarly, SUMO-1-modified chromatin-associated factor SAFB was shown to stimulate initiation by RNA polymerase II. In addition, a deficiency in either SAFB or SUMO-1 abolished the splicing efficiency [242]. Sumoylation was found to be enriched also on chromatin of DNA-encoding exons to favor the protein–protein interactions during RNA processing [229]. SR proteins have also been found to be sumoylated [242,243]. The SF2/ASF was found to interact with UBC9, thus enhancing and promoting sumoylation of other splicing factors [239]. Based on these findings, it can be postulated that sumoylation participates in communication between transcription and splicing.

## 5. Defects in Splicing and Cancer

Splicing is one of the most important processes participating in the regulation of gene expression. However, defects in splicing of genes that regulate cell cycle progression, proliferation, migration, or RNA biogenesis have been observed in cancers and other genetic diseases [244,245,246,247]. Abnormal splice variants have been detected in more than 50% of genetic diseases. Around 15% of point mutations linked with genetic disorders can be directly associated with aberrant splicing [248,249,250]. As such, many human diseases arise due to point mutations that abolish sites of splicing, or stimulate the splicing of cryptic introns [251]. Furthermore, many single nucleotide polymorphism (SNPs) and missense/synonymous SNPs in exons have been detected. These had an impact on enhancer and silencer elements and led to splicing defects [252]. In addition, several mutations that affect *trans*-acting factors give rise to aberrant splicing. For example, in myotonic dystrophies, the muscle blind-like protein MBNL was found to bind to mRNAs with expansions of CUG and CCUG repeats. Sequestering of MBNL abolished its normal function and led to the alternative splicing of various transcripts [253,254,255].

Comparing splicing in normal and malignant cells revealed a significant diversity of their splicing patterns. In particular, mutations in regulatory sequences, spliceosome-associated factors and chromatin modifiers, or altered levels of splicing factors, were frequently detected in cancer cells [256,257]. Furthermore, alternative splicing was found to affect the pathways involved in drug uptake/metabolism of cancer cells [258,259,260,261,262], activation of nuclear receptor pathways [263,264,265], regulation of apoptosis [125,266], or modulation of response to immunotherapy [267]. Moreover, detailed analyses of various tumors revealed that cancer cells contain the splicing variants absent in non-malignant tissues [268,269]. Cancer cells were also shown to cumulate advantageous splicing variants as a result of decreased splicing efficiency [257]. Similarly, tumor cells were found to harbor close to 20% more alternative splicing events than normal tissues. Many splicing changes identified were also linked to single-nucleotide variants of genes with the disrupted mRNA sequences [268,270].

Several studies have pointed out that cancer cells create exonic splicing enhancers or destroy exonic splicing silencers, thereby affecting both oncogene expression and tumor suppressors. For example, mutations within the *TP53* that appeared next to the sites of splicing resulted in retention of introns or activation of a cryptic splice site. This resulted in generation of the frameshifted mRNAs. Such mRNAs are subjects for degradation by the nonsense-mediated decay pathway [271].

It was also found that mutations in the *APC* led to skipping of exons or appearance of new sites of splicing [272,273,274]. Recent studies also revealed numerous novel splice sites creating mutations in cancer cells. These mutations appeared in the genes involved in carcinogenesis, such as *TP53*, *BRCA1*, *ATRX*, *GATA3* and *PARP1* [256,275]. Mutations affecting splicing regulators were also reported in hematopoietic malignancies and are most frequent in patients with myelodysplastic syndromes, myeloid malignancies and chronic lymphocytic leukemia [276,277,278,279]. These mutations were present in the genes coding both core spliceosomal components SF3B1, U2AF1 and SR protein SRSF2, or ZRSR2, which functions in the minor spliceosome analogous to U2AF1 [108,280]. Mutations in these factors were also detected in solid tumors, such as uveal melanoma, pancreatic ductal adenocarcinoma, breast cancers and lung adenocarcinoma [281,282,283,284,285,286,287,288,289].

SF3B1 is known as a member of the U2 snRNP complex, which interacts with p14, U2 snRNA, SF3B3 and PHF5A. RNA-seq analysis of cancer cells identified the K700E and K666N as frequently mutated residues of SF3B1. Mutations in these residues led to creation of cryptic 3′ splice sites [290,291,292,293]. The change in the 3′ splice sites occurred as a result of inaccurate recognition of branch point sequences in the SF3B1 mutant cells [290,294,295,296]. The mutations in SF3B1 occurred within its HEAT repeat domains and led to splicing patterns enriched for alternative 3′ splice site selection [297].

Recent mapping of cancer-associated mutations in SF3B1 suggested that these mutations disturb HEAT repeat domains, thus affecting the binding of SF3B1 with SF3B complex protein p14 and U2AF2 [298,299]. SF3B1 was also affected by the specific hotspot mutations that are associated with specific tumors. One of them, K700E, was detected in myeloid malignancies, breast cancer and pancreatic ductal adenocarcinoma. Additionally, other residues of SF3B1 (e.g., R625, E902 and G742) were also found to be mutated and cancer-specific [270]. Furthermore, hotspot mutations were detected in the splicing factors U2AF1 and SRSF2 of cancer cells. These mutations altered the splice site recognition of these factors in a sequence-dependent manner. Cells with mutated U2AF1 preferred an alternative 3′ splice site selection and exon inclusion, while cells with SRSF2 mutations preferred an altered inclusion of exon. The function of U2AF1 was also affected by hotspot mutations of S34 and Q157 within its zinc finger. On the other hand, point mutations or in-frame deletions at P95 in SRSF2 affected its binding preferences and led to its reduced affinity for G-rich sequences [297,300,301,302,303,304].

Additionally, deregulated expression of splicing factors or spliceosome-associated factors also had an impact on the splicing pattern and led to the development of cancer. For example, SRSF1 was found to be upregulated in many tumor cells. Increased activity of SRSF1 was linked to cell transformation through modulating alternative splicing of several genes, including *RON* and *S6K1* [305,306,307]. Similarly, mutations or misregulated expressions of RBM5, RBM6 and RBM10 have been associated with pathogenesis of lung and other cancers [288,308,309,310,311].

Currently, it has become clear that not only mutations in splicing factors but also their PTMs contribute to dysregulation of splicing processes and might lead to development of cancer [312,313,314,315,316,317]. It was shown that inhibition of the protein kinase SRPK1 reduces phosphorylation of the splicing factors SRSF1 and SRSF2, resulting in significant splicing dysregulation in AML cells. Importantly, the SRPK1-dependent phosphorylation of splicing factor SRSF1 in Y19 was defined as a specific diagnostic marker of some AML patients [318,319,320]. Additionally, the altered splicing was detected in pancreatic ductal adenocarcinoma cells due to enhanced phosphorylation of splicing factor SRSF5 in S250 [321]. Similarly, inhibition of CLKs was found to trigger exon skipping and linked CLKs-dependent phosphorylation of splicing factors to regulation of splicing [322,323]. Furthermore, protein kinase DYRK1A was found to have an important role in splicing homeostasis through specific phosphorylation of splicing factor SF3B1 in T434 [137].

In addition to phosphorylation, the altered ubiquitination and methylation of splicing factors were also found in cancer. It was shown that in MDS cells the overexpressed E3 ligase TRAF6 led to enhanced ubiquitination of hnRNP A1, thus resulting in splicing perturbations [324]. It was also revealed that an aberrant activity of SF3B1 might be attributed to its differential ubiquitination upon oncogenic transformation [325]. In addition, ubiquitin-like PTMs were found to affect the splicing in tumor cells. It was shown that numerous splicing-related factors and proteins, including SF1, SF2, SF3A1, SRSF2, SRSF3 and SRSF7, have altered ubiquitin-like PTMs. This affected their degradation, resulting in significant changes in gene expression and efficacy of splicing [326,327]. Similarly, analyses of tumor cells pointed out the altered methylation of splicing factors SRSF1 and SRSF2 in their arginine residues. This affected their binding capacity to mRNA and other proteins, thus resulting in splicing alterations [328,329].

In summary, the identification of mutations and PTMs of splicing factors and better understanding of their importance for splicing processes may become an attractive strategy to assist in diagnosis or therapy of certain cancers. Given the importance of PTMs, the reliable identification, quantification and characterization of particular PTMs that affect the normal functions of splicing factors is quite challenging. Main obstacles are represented by the heterogeneity of tumor cells. Additionally, the dynamic nature of PTMs makes the identification of cancer-related PTMs even more difficult. However, further advancement in mass spectrometry techniques might allow the analysis of PTMs in patient-derived samples and open up a new horizon for biomarker discovery research [330,331,332,333].

## 6. Conclusions and Future Perspectives

In the past few years, the research on pre-mRNA splicing has experienced great progress, especially with respect to post-translational regulation of factors forming the spliceosome. The improved experimental strategies to characterize the interactomes and post-translational modifications of particular splicing factors when they execute their functions within the spliceosome has brought us much closer understanding of the functional significance of their regulation. Given that most of the splicing factors are post-translationally modified, dysregulation of their PTMs or mutations of the splicing factors themselves may disturb the efficiency and fidelity of splicing, resulting in widespread alterations over the transcriptome and proteome. These imbalances may then alter many cellular processes, leading to the development of multiple diseases, including cancer. Thus, future studies that help to decipher the exact molecular mechanisms participating in regulation of pre-mRNA splicing, including the full understanding of the importance of PTMs for normal function of particular splicing factors, are highly warranted.

## Figures and Tables

**Table 1:** Representative splicing factors, their phosphorylation, selective kinases and function affected.

Splicing Factor	Phosphorylated Residue	Kinase	Function of Splicing Factor	References
PRP28	unknown	SRPK2	spliceosome assembly	[63]
PRP6	S236, S279, T205, T266, T275	PRP4K	spliceosome assembly	[63]
PRP31	S439, S498, S445, S446, S450, S451, T440, T455	PRP4K	spliceosome assembly	[64]
SAP155	T434	DIRK1A	splicing catalysis	[67]
SF1	S20, S80, S82	PKG-I	spliceosome assembly, inhibition of splicing	[72,73]
SRSF1	S201, S208, S209, S198–S218	DYRK1A, CLK1, CLK2, CLK3, CLK4, SRPK1	accuracy of splicing, alternative splicing	[63,74,75,76,100,101,114,132,138]
SRSF2	RS domain	DYRKA1A, SRPK1, SRPK2	cellular localization, protein–protein interactions	[132,137,139]
SRSF6	RS domain, S280, S303, S316	DYRK1A	alternative splice site selection	[140]
SRSF7	RS domain, S217, S223, S232	DYRK1A, PKA	alternative splicing regulation	[114,134,135,139]
SRSF10	unknown	CLK family, SRPK1	splicing repression, alternative splicing	[83,84,85]
SAM68	unknown	BRK, ERK1/2, SRC	splicing regulation, alternative splicing	[91,94,98,117]
SPF45	S48, S62, S202, S204, S222, S266, S288, S291, S405, S418	CLK1 ERK	splice site utilization	[119,120]
U2AF65	unknown	unknown	splice site recognition	[73]

## Data Availability

No new data were created or analyzed in this study.
